# A methodological approach for deep learning to distinguish between meningiomas and gliomas on canine MR-images

**DOI:** 10.1186/s12917-018-1638-2

**Published:** 2018-10-22

**Authors:** Tommaso Banzato, Marco Bernardini, Giunio B. Cherubini, Alessandro Zotti

**Affiliations:** 1 Department of Animal Medicine, Production and Health, University of Padua, Viale dell’Università 16, AGRIPOLIS, Legnaro, 35020 Padua, Italy; 2 Portoni Rossi Veterinary Hospital, Via Roma 57, Zola Predosa, 40069 Bologna, Italy; 3 Dick White Referrals, Six Mile Bottom, Cambridgeshire, CB8 0UH UK

**Keywords:** Convolutional neural network, Meningioma, Glioma, Magnetic resonance imaging, Histopathology

## Abstract

**Background:**

Distinguishing between meningeal-based and intra-axial lesions by means of magnetic resonance (MR) imaging findings may occasionally be challenging. Meningiomas and gliomas account for most of the total primary brain neoplasms in dogs, and differentiating between these two forms is mandatory in choosing the correct therapy. The aims of the present study are: 1) to determine the accuracy of a deep convolutional neural network (CNN, *GoogleNet)* in discriminating between meningiomas and gliomas in pre- and post-contrast T1 images and T2 images; 2) to develop an image classifier, based on the combination of CNN and MRI sequence displaying the highest accuracy, to predict whether a lesion is a meningioma or a glioma.

**Results:**

Eighty cases with a final diagnosis of meningioma (*n* = 56) and glioma (*n* = 24) from two different institutions were included in the study. A pre-trained CNN was retrained on our data through a process called transfer learning. To evaluate CNN accuracy in the different imaging sequences, the dataset was divided into a training, a validation and a test set. The accuracy of the CNN was calculated on the test set. The combination between post-contrast T1 images and CNN was chosen in developing the image classifier (trCNN). Ten images from challenging cases were excluded from the database in order to test trCNN accuracy; the trCNN was trained on the remainder of the dataset of post-contrast T1 images, and correctly classified all the selected images. To compensate for the imbalance between meningiomas and gliomas in the dataset, the Matthews correlation coefficient (MCC) was also calculated. The trCNN showed an accuracy of 94% (MCC = 0.88) on post-contrast T1 images, 91% (MCC = 0.81) on pre-contrast T1-images and 90% (MCC = 0.8) on T2 images.

**Conclusions:**

The developed trCNN could be a reliable tool in distinguishing between different meningiomas and gliomas from MR images.

## Background

Brain neoplasms are a primary concern in adult dogs, with an overall reported prevalence of 4.5% [1]. Treatment options for brain tumours in dogs include symptomatic management, chemotherapy, surgery, radiation therapy, surgery combined with chemotherapy and/or radiation therapy [2]. When symptomatic management or radiation therapy is chosen as the treatment option, histopathological analysis of the lesions is usually not performed and the diagnosis is based only on interpretation by the imaging expert [3]. Although some imaging features may be used to increase or decrease suspicion of a particular tumour type, the distinction between meningeal-based and intra-axial lesions may occasionally be challenging [4]. Meningiomas and gliomas account for most of the total primary brain neoplasms in dogs [1], and differentiating between these two forms is mandatory in choosing the correct therapy.

The role of diagnostic imaging grows progressively more important as the demand for high quality veterinary care constantly increases. In such a scenario, a thorough standardisation in interpretation of diagnostic images becomes ever more desirable. The possible applications of a texture analysis-based approach on other diagnostic imaging techniques such as MRI [5] or computed tomography [6] have only seldom been investigated in veterinary medicine. On the other hand, several studies exploring the use of texture analysis to establish the relationship between ultrasonography and pathology have been published [7–13]. The main purpose of these studies was to overcome the inherent limitations of ultrasonography in identifying subtle changes in the appearance of parenchymal organs (mainly kidney and liver) caused by degenerative pathologies.

In the present work we have tried to take advantage of CNNs in the extraction and analysis of complex data patterns in order to distinguish between meningiomas and gliomas in pre- and post-contrast T1 images and T2 images. Furthermore, we have developed an image classifier, which could be prospectively used in a clinical scenario, to predict whether a lesion is a meningioma or a glioma; such a classifier is based on the combination of CNN and MRI sequence displaying the highest accuracy.

## Materials and methods

### Cases selection

The databases of two different institutions [Portoni Rossi Veterinary Hospital (Institution 1), Zola Predosa, Italy; Dick White Referrals, Six Mile Bottom, UK (Institution 2)] were retrospectively searched between January 2011 and January 2018 for dogs having an MRI scan showing an intracranial space-occupying lesion and a final histopathological diagnosis of either meningioma or glioma. No a-priori selection based on the histopathological classification of the lesions was made at this stage.

### MR imaging

The MRI scans were performed with a 0.4 T open-type permanent magnet (Hitachi Aperto, Hitachi Medical Corporation, Japan) at Institution 2, and with a 0.22 T open-type permanent magnet (MrV, Paramed Medical Systems, Genova, Italy) at Institution 1. Different imaging protocols were used at the two institutions. Only MRI scans including a T2W fast spin-echo series (repetition time, 13 to 120 ms; echo time, 290 to 7790 ms; matrix, 512 × 512 pixels) and pre- and post-contrast (gadolinium-based medium) T1W spin-echo series (repetition time, 13 to 26 m; echo time, 462 to 880 ms; matrix, 512 × 512 pixels) were included in the study. All images were acquired with 3- to 5-mm slice thickness with a 10% gap, while the signal-to-noise ratio was improved using 2 to 4 averages for each acquisition.

### Dataset preparation

All the MRI studies were exported in a .jpg format from the original digital imaging communication in medicine (DICOM) format. Pre- and post-contrast T1 and T2 sequences were included in the study. Images belonging to different imaging sequences were analysed separately. Dorsal, sagittal and transverse scans were selected to increase the number of available images. All lesion-containing images were divided into two different folders based on the final histopathological diagnosis (meningioma or glioma). Thereafter, the images were cropped so that only the lesion and a small portion of the surrounding tissues were included. Lastly, the images were resized, using a photo editing program (PhotoshopCC, Adobe Sytems Incorporated, USA), to a 224 × 224-pixel format to match the CNN requirements.

### Deep learning model

Due to the limited size of our database, we retrained a pre-trained CNN called GoogleNet [14] on our images, a process called “transfer learning”. The built-in MATLAB (MATLAB and Statistics Toolbox Release 2017b, The MathWorks, Inc., Natick) toolbox for neural networks was used for the experiment. GoogleNet was trained on a large-scale image database [ImageNet database (www.image-net.org)] comprising approximately 1.2 million everyday images belonging to 1000 different categories. GoogleNet is an extremely deep neural network (it comprises 144 different layers) and is composed of several layer types with specific functions. An in-depth description of the structure of GoogleNet is beyond the purposes of this paper but a general description of how CNNs work is useful to its clarity. The basic components of a CNN are: convolutional layers, pooling layers and dense layers. Convolutional layers extract a large number of features from the images and create maps of the distribution of these features throughout the image. Deeper convolutional layers are able to detect more complex features (Fig. 2). Pooling layers are used to reduce data volume, decreasing the size of the feature maps while retaining the most important information. The dense layers are the classification layers and are the equivalent of a classical artificial neural network; a set of interconnected neurons that analyse an input and generate an output to make predictions on new data.

The features (along with their weights and biases) derived from the ImageNet database were then adjusted on the new dataset to predict the labels of the new images (transfer learning).

### Evaluation of the classification performance of GoogleNet in the different MRI sequences

To prevent overfitting (i.e. poor generalisation performance), the images in the dataset were randomly divided into a training set, a validation set and a test set, respectively comprising 70%, 15% and 15% of the images. The validation set was used to fine-tune the network parameters and the test set was used to test network accuracy. If only a training set and a test set are used, there is a high risk of over-adapting the network to the test data, with consequent poor generalisation performance (overfitting). The network parameters were set as follows: LearnRateSchedule = piecewise, MaxEpochs = 120. An early stopping function was used to further prevent overfitting; if accuracy in the validation set stopped increasing for five consecutive epochs (an epoch is a complete iteration of the network throughout the training set), the learning phase was terminated [15]. Accuracy of a CNN is measured by the loss (or cost) function: the loss function measures the difference between the CNN output and the real label of the data. The lower the cost function value, the higher the network performance. When the loss stops decreasing, the CNN has reached the optimal solution (meant as the best possible accuracy given the network, dataset and settings) for the classification problem. The learn rate defines how large the network steps to reach the optimal solution are; if the steps are too big the optimal solution may be skipped, if the steps are too small the network could take an unreasonable amount of time to train. We programmed the network to adapt the learn rate to the learning process so that the learn rate decreased the closer the network got to the optimal solution. Classification accuracy was then displayed as the percentage of correctly labelled images in the test set and as a confusion matrix for the real and predicted image category. In order to account for the random distribution of the images in the training, validation and test sets, the analyses were repeated five times.

A cross-classification table method was used to calculate the accuracy of the trained classifier. Accuracy was calculated as the percentage of correctly classified cases. To compensate for the different distribution of the cases between the two classes (the total number of meningiomas was more than twice that of gliomas), additional metrics of accuracy, such as sensitivity, specificity, Cohen’s Kappa (CK), and the Matthews correlation coefficient (MCC) [16], were calculated. The data are reported as median with the limits of the overall range.

### Development of the trained classifier (trCNN)

To develop and test our trained classifier we asked one of the authors (MB, board- certified neurologist) to select five cases in which, based on the imaging reports, lesion location (intra- or extra- axial) made it difficult to assess. Ten images (five belonging to meningioma cases and five to glioma cases) were selected and excluded from the database used to retrain the network. GoogleNet was then retrained on the entire set of images (minus the ten selected images) (trCNN) and later used to predict the labels for the 10 previously excluded images.

## Results

Eighty cases were included in the study. Twenty-four cases had a final diagnosis of glioma (Institution 1 *n* = 14; Institution 2 *n* = 10) and 56 of meningioma (Institution 1 *n* = 23; Institution 2 *n* = 33). Forty-five meningioma cases included in the present study (Institution 1 *n* = 18; Institution 2 *n* = 27) were also part of a previous study (Banzato et al., 2017) on texture image analysis. Complete results of the histopathological analysis are reported in Table 1. Six of the 56 meningioma cases were discarded because the lesions were completely cystic and only an insufficient amount of tissue was available for analysis.

**Table 1:** Complete histopathological results of the cases included in the study

Histopathological type	Number of cases
Gliomas (*n* = 24)	
Oligodendroglioma	12
Astrocytoma	8
Glioblastoma	3
Oligoastrocytoma	1
Meningiomas (*n* = 56)	
Papillar	11
Transitional	9
Atypical	6
Meningothelial	4
Fibroblastic	4
Psammomatous	3
Syncytial	3
Lipomatous	3
Meningoendothelial	3
Chordoid	2
Anaplastic	2
Other (biphasic, cystic, malignant, microcystic, osteoid, vacuolar, vascular)	6

**Fig. 1:**
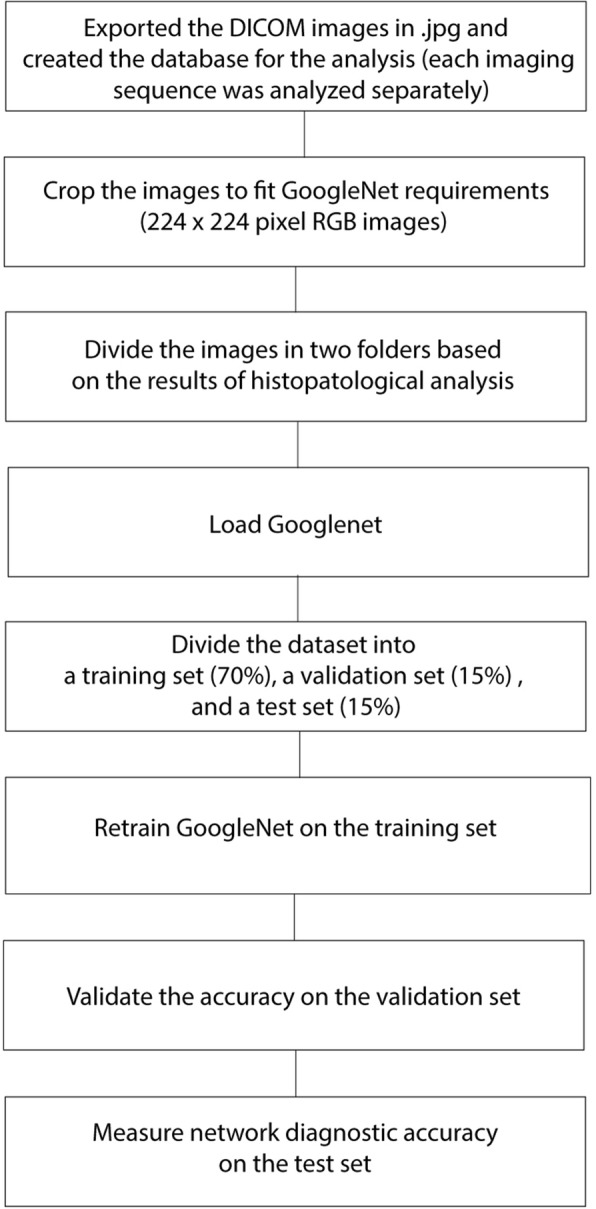
Workflow used for the experiment

**Fig. 2:**
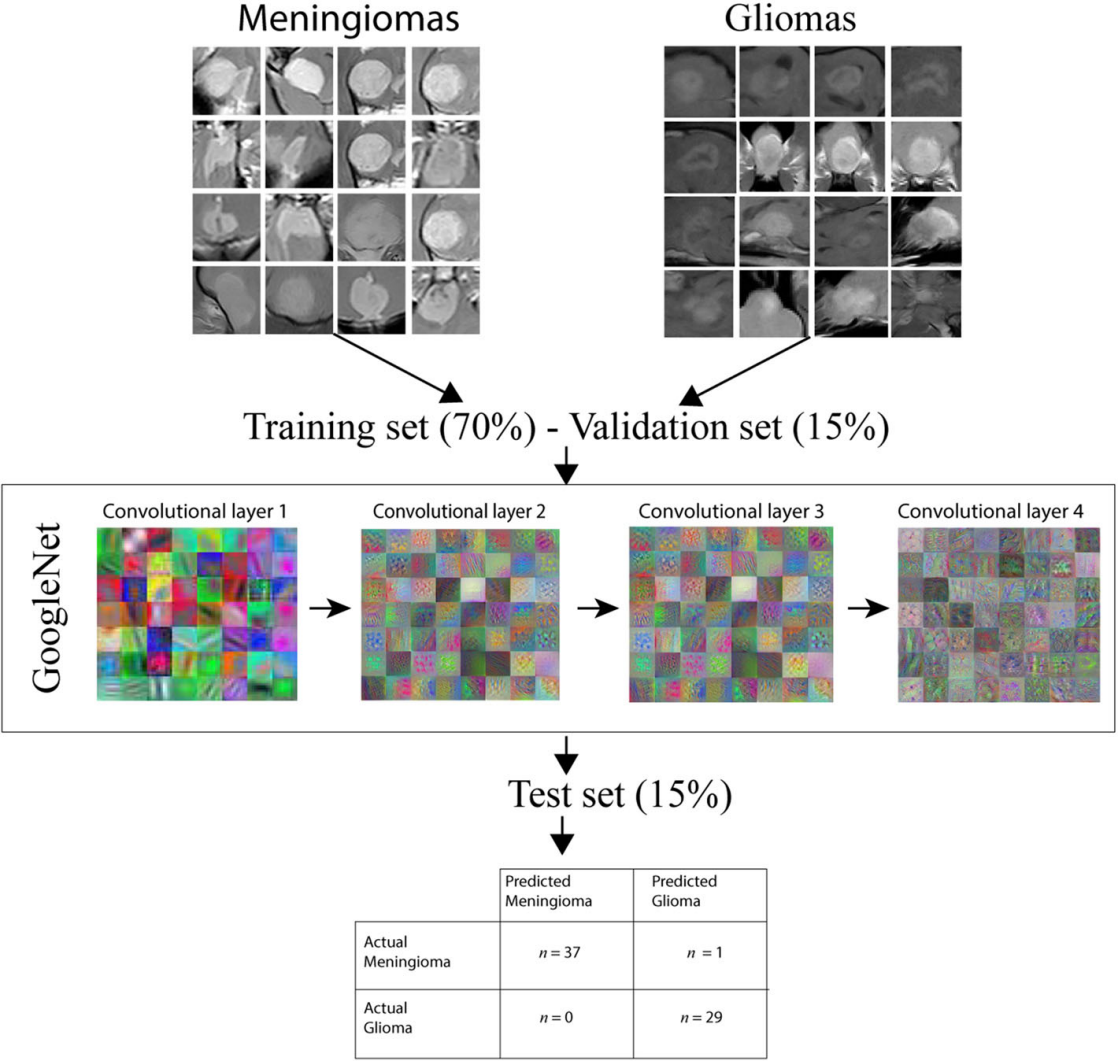
Simplified representation of the analytical method used in the experiment and analytical output. The images are divided into two folders based on the results of the histopathological analysis. Thereafter, the dataset is divided into a training, a validation and a test set. The training and the validation sets are used for the transfer-learning procedure with GoogleNet. A schematic and simplified representation of the output of the first convolutional layers is reported. Please note that the features represented become more complex during convolutions. Lastly, the retrained GoogleNet convolutional deep neural network is used to predict the labels for the test set. A confusion matrix is generated as a final output. *n* = number of images

The complete CNN workflow is reported in Fig. 1. A schematic representation of the analytical procedure, along with the analysis output, is reported in Fig. 2.

GoogleNet displayed the best performance on post-contrast T1 images, with a 94% accuracy (range: 89–98%). Sensitivity was 0.94 (range: 0.87–0.97), specificity was 0.94 (range 0.82–1), CK was 0.87 (range: 0.78–0.97), and MCC was 0.88 (range: 0.78–0.97).

The classification performance of GoogleNet on pre-contrast T1 images was lower, with a 91% accuracy (range: 88–92%). Sensitivity of 91% (range: 88–100%), specificity of 91% (range: 88–96%), CK of 0.81 (range: 0.75–0.86) and MCC of 0.81 (range: 0.75–0,86) were recorded.

GoogleNet had the poorest performance on T2W images, with a 90% (range: 89–93%) accuracy. Sensitivity was 89% (range: 83–96%), specificity was 91% (range: 83–97%), CK was 0.8 (range: 0.77–0.85) and MCC was 0.8 (range: 0.77–0,85).

Lastly, the trCNN correctly classified all the 10 images (from 3 glioma and 2 meningioma cases) that had previously been excluded from the database.

## Discussion

Several image analysis techniques have been proposed both in human [17] and veterinary medicine [10] in recent years. One of the main advantage of deep learning among other image-analysis techniques (such as texture analysis) is that deep learning algorithms can be trained directly on the images and, once developed, can be applied to new images to make predictions [18]. A specialised class of deep-learning architectures, the so-called convolutional neural networks (CNNs), are considered the state-of-the-art algorithms for image analysis and classification [19]; a substantial number of different applications are being developed in medical imaging for structure detection, image segmentation, and computer-aided diagnosis [20]. Deep learning is also gaining popularity in medical imaging for other tasks such as: the automated creation of study protocols, improving image quality while decreasing radiation dose in CT; improving image quality and reducing scan time in MRI; plus many others [21]. The increasing availability of computers with great computational powers, as well as the scope to easily create and share large datasets, are acting as boosters for the development of deep-learning-based applications in the medical-imaging field, and the routine use of some applications assisting the radiologist’s decision-making process is likely to be seen in the near future [22]. Recently, the possibility of using deep learning to detect degenerative liver disease in canine patients from ultrasonographic images has been explored [23].

GoogleNet displayed a very high accuracy on all the imaging sequences (more than 90% of the images were correctly labelled) in discriminating between meningiomas and gliomas, suggesting that the use of transfer learning was an appropriate solution to our classification problem. In testing our trCNN, the test-cases were selected based on the opinion of MB (co-author, board-certified neurologist), since one of the aims of this study was to evaluate trCNN performance in those cases that resulted as challenging for expert radiologists. In particular, in our experience, it is far more common for a glioma to resemble an extra-axial neoplasm rather than for a meningioma to resemble an intra-axial lesion. Prospectively, use of the CNN developed in this study might help the clinician in the distinction between intra-and extra axial lesions.

The most important limitations of this work are its relatively low number of cases and the imbalance between glioma (24) and meningioma cases (56). However, it is the authors’ opinion that such an imbalance did not act as a major limitation, due to the high classification accuracy displayed by the trCNN. GoogleNet classification performance was carefully evaluated using metrics of accuracy, such as MCC, which were specifically developed to assess the performance of a classifier on heavily imbalanced databases. In particular, MCC takes values in the interval [− 1, 1], with 1 showing a complete agreement, − 1 a complete disagreement, and 0 showing that the prediction was uncorrelated with the ground truth [24]. The MCC of GoogleNet applied on post-contrast T1 images was 0.88 (range: 0.78–0.97), indicating a very high agreement between the real and the predicted histopathological classes of the images.

Based on the data reported in Table 1, it is remarkable that the model proposed here showed excellent classification results despite the intrinsic variability of histological subtypes in both gliomas and meningiomas. Further studies, preferably including a larger number of patients from various institutions, are needed to determine the real generalisation performance of our trCNN.

Another important limitation is that, with the model we proposed, only two histopathological classes of brain tumours were included in the study and the trCNN had to classify each lesion as meningioma or glioma regardless of the actual nature of the lesion. However, the aim of this methodological study is not to propose a ready-to-use clinical test but to explore, retrospectively, the capacity of CNNs to distinguish between the two most common primary brain tumours in the dog. The excellent classification results achieved by our trCNN suggest that CNNs could become useful tools for both neuro-radiologists and clinicians in planning the correct therapeutic protocol. The next step towards development of a routine clinical application should include more categories of brain disease (both neoplastic and non-neoplastic) to further test the accuracy of deep learning in an actual clinical scenario.

## Conclusions

The results reported in the present study suggest that CNNs could be a reliable tool in distinguishing between different meningiomas and gliomas from MR images. Further studies, possibly including a larger number of cases and histopathological categories, are required to determine the performance of CNNs in a clinical scenario.

## Abbreviations

CK: Cohen’s Kappa
CNN: Convolutional neural network
MCC: Mathews correlation coefficient
MR: Magnetic resonance
trCNN: Trained convolutional neural network
